# A Mononuclear Iron(II) Spin-Crossover Molecule Decorated by Photochromic Azobenzene Group

**DOI:** 10.3390/molecules27051571

**Published:** 2022-02-27

**Authors:** Jiang-Zhen Qiu, Yong You, Ye Yu, Zhuo-Fan Chen, Cheng-Jie Guo, Yi-Ling Zhong, Wei-Quan Lin, Xu-Gang Shu

**Affiliations:** 1 College of Chemistry and Chemical Engineering, Zhongkai University of Agriculture and Engineering, Guangzhou 510225, China; yong_youyy@163.com (Y.Y.); tim_yeyu@126.com (Y.Y.); zhuofan_chen01@126.com (Z.-F.C.); chengjie_guo@126.com (C.-J.G.); 2 Guangzhou Key Laboratory for Clean Energy and Materials, School of Chemistry and Chemical Engineering, Guangzhou University, Guangzhou 510006, China; zyl12342022@163.com

**Keywords:** spin crossover (SCO), azobenzene, *cis–trans* photoisomerization

## Abstract

Aiming at constructing photoresponsive spin crossover (SCO) behavior, herein we designed a new ligand Abtz (Abtz = (*E*)-*N*-(4-((*E*)-phenyldiazenyl)phenyl)-1-(thiazol-4-yl)methanimine) which was decorated by a photochromic azobenzene group. Based on this photochromic ligand, a mononuclear Fe(II) SCO molecule [Fe(Abtz)_3_](BF_4_)_2_·(EAC)_2_ (**1**, EAC = ethyl acetate) was successfully synthesized and showed a complete one-step SCO behavior. Under continuous UV light and blue-light exposure, the *cis–trans* photoisomerization of both ligand Abtz and compound **1** in the liquid phase was confirmed through UV–Vis spectra. Moreover, the ^1^H-NMR spectra of Abtz reveal a *trans–cis* conversion ratio of 37%. Although the UV–Vis spectra reveal the photochromic behavior for **1** in the solution phase, the SCO behavior in the liquid state is absent according to the variable-temperature Evans method, suggesting the possible decomposition. Moreover, in the solid state, the *cis–trans* photoisomerization of both Abtz and **1** was not observed, due to the steric hindrance.

## 1. Introduction

Complexes [1,2,3,4,5,6] of 3d^4~7^ transition metal ions have attracted intense interest from scientists due to their fascinating multi-functional properties on nonlinear optical [7], magnetism [8], conductivity [9,10,11,12] and mechanical properties [13], which result from the d-orbital electron rearrangement between the low-spin (LS) and high-spin (HS) electronic configurations under external stimuli, e.g., heat, optical and pressure. This ability of spin crossover (SCO) compounds to tune their functional properties shows potential applications in the fields of information storage, sensors and actuators. Therefore, controlling SCO properties by light irradiation is very appealing, because of its low-cost, accurate and convenient features. For example, the light-induced excited spin-state trapping [2,3,14] (LIESST) shows that the metastable HS state could be captured through the photoexcitation of the LS state in SCO compounds at low temperatures. However, a very low temperature is necessary for stabilizing the photoexcited states, hindering further application.

In this context, the integration of photoactive organic ligand into SCO systems gives an alternative way to control the SCO behavior in ambient conditions. For example, Zarembowitch et al. put forward the ligand-driven light-induced spin crossover (LD-LISC) approach [15,16,17,18], showing the possibility to switch the spin state through generating different ligand fields around the SCO center from the two isomeric forms of photoreactive ligands. Based on this strategy, a series of SCO compounds decorated by photochromic groups, showing *cis–trans* photoisomerization behavior [19,20], intramolecular photocyclization [21,22,23,24] and photochemical [2 + 2] cycloaddition [25,26], which were reported in recent years, and the corresponding photoresponsive SCO behaviors were further studied in the solid or liquid state. Moreover, a porous Hofmann-type SCO framework encapsulating *trans*-azobenzene photochromic guests [27] was reported by Tong et al. recently. Under 365 nm irradiation, about 20% *cis*-isomer is obtained and could be reversed to *trans*-isomer under dark or visible-light exposure. Although azobenzene units are not directly decorated around the SCO center, it is found that about 22% SCO center undergo HS↔LS conversion as a result of the framework expansion during the *cis–trans* photoisomerization, showing guest-driven light-induced spin change (GD-LISC).

Inspired by the potential synergy of ligand photoreactivity and SCO system, herein we report a mononuclear Fe(II) molecule [Fe(Abtz)_3_](BF_4_)_2_·(EAC)_2_ (**1**, Abtz = (*E*)-*N*-(4-((*E*)-phenyldiazenyl)phenyl)-1-(thiazol-4-yl)methanimine; EAC = ethyl acetate), which displays a one-step SCO behavior, based on a photochromic chelating ligand Abtz. Under continuous UV and blue-light irradiation, the reversible *cis–trans* photoisomerization of the azobenzene group of Abtz ligand in liquid is observed with a conversion ratio of 37%. However, due to the possible decomposition in the liquid state and steric hindrance in the solid state, the *cis–trans* photoisomerization of **1** is not observed in both liquid and solid states, indicating an inert LD-LISC behavior.

## 2. Results

### 2.1. Magnetic Properties

**Scheme 1:**
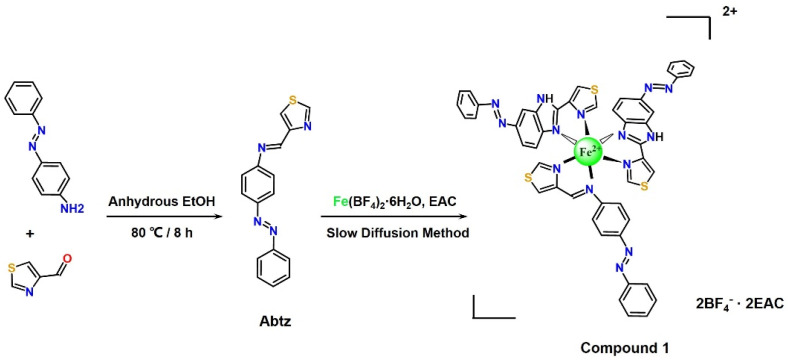
Synthesis procedure of Abtz ligand and **1**.

**Figure 1:**
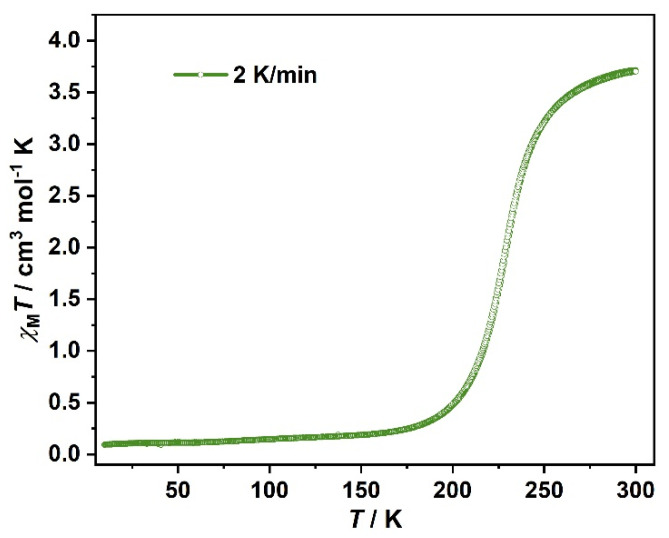
Temperature-dependent molar magnetic susceptibility, *χ*_M_*T*, products for **1** under 2 K/min sweeping rates.

Red block crystals of **1** were obtained through the slow diffusion method between Abtz and Fe(BF_4_)_2_·6H_2_O in ethyl acetate (EAC) solution (see Scheme 1 and experimental section). The purity of the microcrystalline samples for **1** was confirmed by elemental analysis (EA) and powder X-ray diffraction (PXRD). The magnetic susceptibility measurements were performed for the crystalline samples of **1** in the temperature range of 10–300 K to study the SCO behavior (Figure 1). At 300 K, the *χ*_M_*T* of **1** is 3.7 cm^3^ mol^−1^ K, corresponding to a pure HS state for Fe(II) center with *S* = 2, *g* = 2.22. Upon cooling with a 2 K/min sweep rate, it was found that the *χ*_M_*T* product underwent a relatively abrupt decreasing to the value of 0.19 cm^3^ mol^−1^ K at 150 K and stayed almost unchanged at the lower temperature region, indicating a complete HS→LS conversion. Further warming revealed that the curve progression of *χ*_M_*T* value is the same as the cooling mode, showing a one-step SCO behavior without thermal hysteresis. According to the *χ*_M_*T* vs. *T* data, the *T*_1/2_, which corresponds to a mixture HS_0.5_LS_0.5_ intermediate state, was calculated to be 229 K for **1**.

### 2.2. Structural Characterization

**Figure 2:**
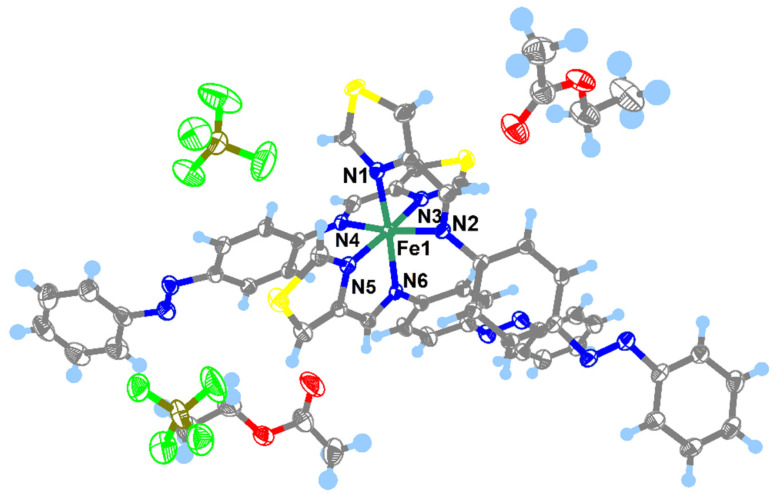
Asymmetric unit of Compound **1** at 150 K with thermal ellipsoids at 50% probability. Color code: Fe, dark green; S, yellow; N blue; F, green; C, gray; O, red; B, brown; H, light blue.

According to the magnetic data, the single-crystal X-ray diffractions of **1** were further performed at 150, 229 and 300 K. Compound **1** kept crystallizing in the triclinic space group *P*-1 at all measured temperatures (Supplementary Materials Table S1). Therefore, only the structure at 150 K for **1** is described in detail. As shown in Figure 2, the asymmetric unit of **1** consists of one Fe(II) ion, three crystallographically equivalent Abtz ligands, two BF_4_^−^ anions and two EAC solvent guests, giving the formula [Fe(Abtz)_3_](BF_4_)_2_·(EAC)_2_. Each Fe(II) ion in **1** is chelated by three Abtz ligands, leading to the mononuclear [Fe(Abtz)_3_]^2+^ molecule. The unit cell of 1 consists of two [Fe(Abtz)_3_]^2+^ molecules, which are related by the inversion center (Supplementary Materials Figure S1). The photochromic azobenzene groups in Abtz ligands show a *trans* form. The six Fe–N bond lengths and angles (Table 1 and Table S2) indicate a distorted [FeN_6_] octahedron coordination. The average Fe–N bond lengths at 150, 229 and 300 K are 1.997, 2.091 and 2.191 Å, corresponding to the LS, LS_0.5_HS_0.5_ and HS state for the Fe(II) ions, respectively, which are in agreement with the magnetic data.

Numerous supramolecular interactions were observed in **1**. It was found that each [Fe(Abtz)_3_]^2+^ molecule interacted with three adjacent mononuclear Fe(II) molecules through offset face-to-face *π*…*π* interactions and edge-to-face C-H…*π* interactions (Supplementary Figure S2). BF_4_^−^ ions and EAC solvent guests filled in the space between the mononuclear Fe(II) molecules and were stabilized through the intermolecular interaction, such as F···H and O···H contacts. As a result, no void was observed in the structure of **1**. The further thermogravimetry (TG) analysis of **1** indicated that the EAC solvent guests started desolvating above 100 °C, showing high thermal stability due to the close packing structure for **1**. Based on the comparison of resulting fingerprint plots from Hirschfeld surface [28] analysis at 300 and 150 K, which facilely visualizes the close interactions between the asymmetric units in **1**, a shifting of the whole pattern from the upper right corner toward the lower-left corner was found, indicating that the distances from the surface to the nearest internal (di) and external (de) atoms were both shortened due to the shrinkage of the lattice during the HS→LS conversion and cooling (Supplementary Figure S3). Moreover, an apparent change of the four F atoms from BF_4_^−^ ions was observed by comparing the asymmetric units for **1** at 300 and 150 K (Supplementary Figure S4), indicating the rotation of BF_4_^−^ ions during the SCO.

### 2.3. ^57^Fe Mössbauer Spectra

**Figure 3:**
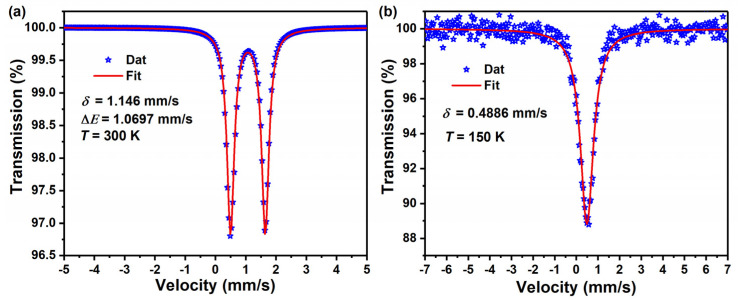
^57^Fe Mössbauer spectra for **1** measured at *T* = 300 K (**a**) and 150 K (**b**). Experimental data: empty stars. Calculated data: red full lines.

According to the magnetic and crystallographic data, the transmission ^57^Fe Mössbauer spectra (Figure 3) for **1** were further collected at 300 and 150 K, with the aim to more deeply understand the SCO development. It is found that the spectrum of **1** measured at 300 K shows only one doublet (Figure 3a) with the value of the isomer shift (*δ*) and quadrupole splitting (Δ*E*_Q_) lying in the interval expected for Fe(II) in the HS state. At 150 K, only one singlet (Figure 3b) with an isomer shift value of *δ* = 0.4886 mm/s is observed, confirming the pure LS state for the Fe(II) centers. These results corroborate nicely with those obtained from the crystallographic data, as well as the magnetic analysis.

### 2.4. Photo-Isomerization Study for Abtz and **1** in Solution

**Figure 4:**
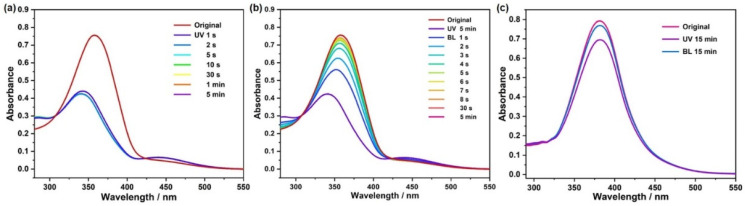
Time-resolved UV–Vis absorption spectra upon (**a**) 365 nm UV irradiation for Abtz; (**b**) continuous 450 nm blue-light irradiation for Abtz; (**c**) 365 nm UV irradiation and 450 nm blue-light irradiation for **1** in ethyl acetate solution.

Since Abtz is decorated by a photochromic azobenzene group, its potential reversible *trans*–*cis* photoisomerization was studied in both liquid and solid states. As seen in Figure 4a, the UV–Vis spectra of Abtz in ethyl acetate show an intense absorption peak at 358 nm in its *trans* form, which is attributed to the *π*–π* transition of azobenzene group. Under the 365 nm UV-light irradiation at room temperature, the 358 nm *π*–π* band underwent a fast decreasing and blue shift to 345 nm within 1 min. Concomitantly, the absorption band at 440 nm corresponding to *n*–π* transition of *cis*-azobenzene was increased. Further prolonging irradiation time did not change the UV–Vis spectra, indicating the instantaneity *trans–cis* photoisomerization reaches photostationary state (PSS). Additionally, the reverse *cis-*to*-trans* conversion is obtained through blue-light irradiation. As shown in Figure 4b, under 450 nm irradiation of blue light within 5 min, the intensity of *π*–π* band for the *trans* form increase and red shift from 342 to 357 nm, showing a *cis→trans* conversion.

**Figure 5:**
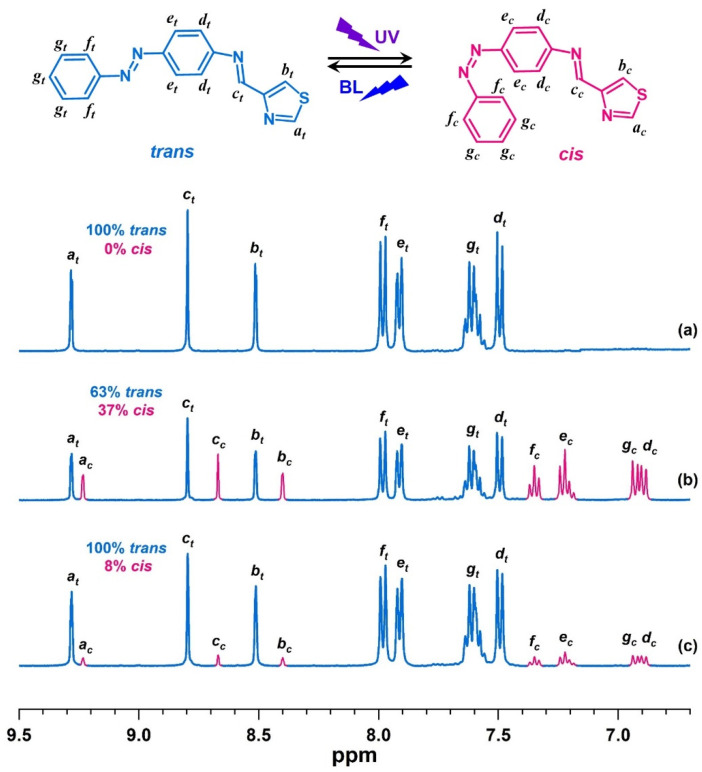
^1^H NMR spectra and their change of Abtz in methyl sulfoxide-*d*6 were obtained (**a**) before irradiation (0% *cis*); (**b**) after UV irradiation (37% *cis*) and (**c**) after continuous blue-light irradiation of b (8% *cis*).

The ^1^H NMR spectra of Abtz in DMSO-*d*_6_ under different wavelength light irradiation were performed to quantitatively analyze the *cis–trans* photoisomerization ratios of Abtz ligand. Before irradiation, the ^1^H NMR spectrum of Abtz reveals seven CH protons signals in the 9.4–7.4 ppm range, which correspond to all *trans* form (Figure 5a, all marked blue). Upon 365 nm light irradiation to reach PSS, new signals assigned to *cis*-Abtz were observed, giving the *cis* molar ratio of 37% (Figure 5b, all marked pick). Further 450 nm irradiation leads to a decreasing of the *cis*-form signals with a ratio of 7% (Figure 5c), showing the reverse *cis-*to*-trans* conversion.

The UV–Vis absorption spectrum of **1** in ethyl acetate solution before and after different wavelength light irradiation was also studied (Figure 4c). One intense absorption peak at 385 nm was observed and should be attributed to the *π*–π* transition of azobenzene groups. Upon irradiation at 365 nm to **1** for 15 min, an obvious decrease in the *π*–π* band was observed, indicating the *trans-*to*-cis* photoisomerization for the azobenzene groups in **1**. Moreover, the spectra changes could be recovered through further 450 nm irradiation for 15 min, thus revealing the reversible *trans–cis* photoisomerization for **1** in the solution.

### 2.5. Magnetism Study for 1 in Solution Phase

**Figure 6:**
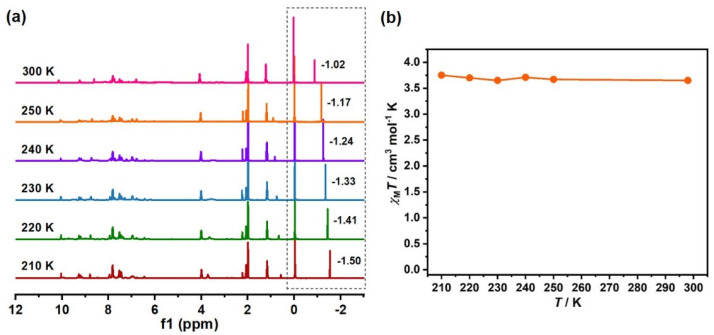
(**a**) ^1^H NMR spectra for **1**, using variable Evans method in the temperature range of 300–210 K. (**b**) Calculated *χ*_M_*T* vs. *T* plot for **1** in the temperature range of 210–300 K.

Considering the reversible *trans–cis* photoisomerization for **1** in solution, the magnetization for **1** in the liquid state was further estimated according to the paramagnetic shift of the reference tetramethylsilane (TMS) signal from the ^1^H NMR spectra, using the variable-temperature Evans method [29] in the range of 210–300 K (Figure 6a). To this end, a coaxial NMR tube containing the acetone-*d*6 and DMF-*d*_7_ (10/1) mixture solution of **1** (*c* = 2 × 10^−2^ mol·L^−1^) with 5% vol.-TMS at the insert tube and the solute with the reference at the outer tube. The susceptibility, *χ*_M_, was calculated according to the difference of the chemical shifts, Δ*f*, of the TMS proton signals in the inner and outer tubes. Diamagnetic corrections were determined from Pascal’s constants [30]. For **1**, although the SCO behavior was observed in the solid state, the *χ*_M_*T* for **1** in the solution maintains around 3.7 cm^3^ mol^−1^ K in the whole temperature region of 210–300 K (Figure 6b), indicating the absence of the SCO behavior. Considering the potential coordination ability of DMF solvent, it is rational to speculate the decomposition of **1** in solution and a change of the coordination environment of Fe(II) centers, thus causing the HS state and hindering a further study of the LD-LISC behavior in solution phase. The attempt on changing of other solvents without coordination ability, such as chloroform-*d*7 and ethyl acetate-*d*8, was failed due to the very low solubility (<0.01 m mol·L^−1^) for **1**.

### 2.6. Photo-Isomerization Study for Abtz and **1** in Solid

**Figure 7:**
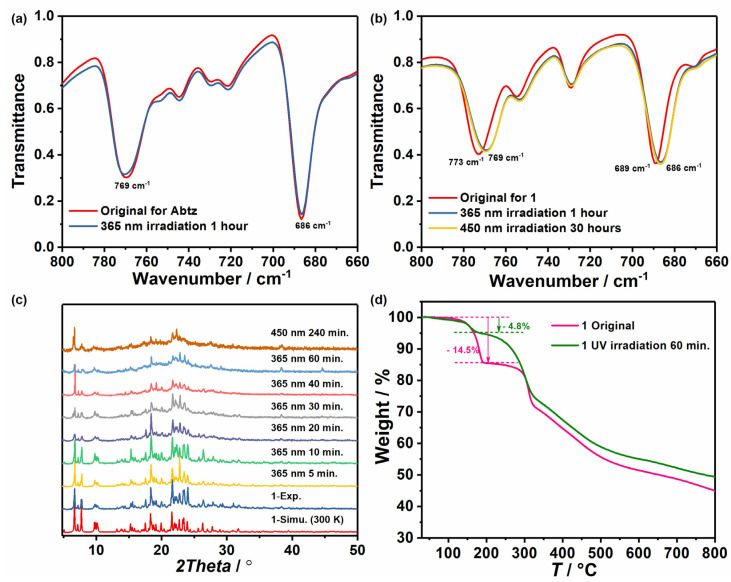
(**a**) IR spectra of Abtz in KBr pellet before and after irradiation with the UV and blue lamp. (**b**) IR spectra of **1** in KBr pellet before and after irradiation with UV lamp. (**c**) PXRD patterns of **1** before and after UV and blue-light irradiation at a different time. (**d**) TG curves of **1** before and after UV irradiation for 1 h.

The UV-light exposure of both Abtz ligand and **1** in solid state was also performed. Solid-state IR spectroscopy was used to monitor this irradiation process. For Abtz ligand, two IR peaks at 769 and 686 cm^−1^ are observed and should be ascribed to the coupling of *γ*(CH) and *γ*(ring) modes in *trans*-azobenzene (Figure 7a). After UV irradiation of 365 nm for 1 h, the IR spectra remain unchanged, showing that the effective photoconversion of Abtz in the solid state is hampered by steric hindrance and intermolecular interactions. For compound **1**, the two peaks, which correspond to *trans*-azobenzene, are observed at 773 and 689 cm^−1^ (Figure 7b). After the 365 nm irradiation of 1 h, the slight red-shifting of these peaks was observed. However, no noticeable intensity change was observed, and the new peak, which was assigned to the coupling of *γ*(CH), *δ*(NNC) and *δ*(ring) modes of *cis-*azobenzene, at around 701 cm^−1^ [31], was not observed.

What is more, the IR spectra were maintained unchanged under further blue-light irradiation. For further studying this behavior, we performed the PXRD measurement for compound **1** under different UV irradiation times (Figure 7c) and found that the crystalline phase gradually disappeared with the lengthening of the irradiation time. Moreover, the thermogravimetric (TG) analysis of the irradiated samples of **1** (Figure 7d) only shows 4.8% weight loss at the first weight loss step, which is lower than the calculated 13.7% weight loss for two EAC molecules, further confirming the desolvation of about 70% EAC molecules during irradiation and thus leading to the molecule packing rearrangement in the structure. Therefore, according to the PXRD and TG analysis, the change of IR spectra for **1** during the UV irradiation is more likely attributed to the change of the structural packing, due to the loss of solvent EAC guests.

## 3. Materials and Methods

### 3.1. General Remarks

All used materials were obtained from commercial sources, without further purification. All experiments for *trans–cis* photoisomerization were conducted at room temperature. The *trans*-to-*cis* photoisomerization experiments were performed by using a UV LED lamp (10 W) with *λ* = 365 nm, and the *cis*-to-*trans* photoisomerization experiments were performed by using a Blue LED lamp (5 W) with *λ* = 450 nm. Powder X-ray diffraction (PXRD) data were collected on a Rigaku SmartLab diffractometer (Tokyo, Japan) provided with a rotating anode (Cu Kα1 radiation, *λ* = 1.5406 Å) in a *2θ* range of 4–50°. The UV–Vis absorption spectra were recorded on a Perkin Elmer Lambda 950 spectrophotometer (Waltham, MA, USA), and the IR spectra were collected within KBr tablets on a Nicolet 6700 FTIR spectrometer (Waltham, MA, USA) in the range of 400–4000 cm^−1^. The ^1^H NMR spectra were recorded on a Bruker advance III 400 MHz spectrometer (Fällanden, Switzerland). The thermogravimetric (TG) test was carried out on NETZSCH TG 209F1 Libra (Selb, Germany) from 30 to 800 °C, with a heating rate of 10 °C min^−1^, in a nitrogen atmosphere. A Vario EL Cube elemental analyzer (Hanau, Germany) conducted elemental analysis for C, H and N. ^57^Fe Mössbauer spectra of **1** were collected in the transmission geometry, using a Mo Mössbauer spectrometer (Prague, Czech) operating at a constant acceleration mode and equipped with a 50 m Ci ^57^Co (Rh) source. Magnetic susceptibility measurements were measured on powder samples, using a Quantum Design MPMS-XL SQUID susceptometer (San Diego, CA, USA). at 10−300 K, with a rate of 2 K·min^−1^ under an applied dc field of 1000 Oe. The Pascal constants were used for the diamagnetic corrections.

### 3.2. Crystal Structure Determination

Single-crystal X-ray diffraction data were collected on a Bruker D8 QUEST diffractometer (Karlsruhe, Germany) with Mo-Kα (*λ* = 0.71073 Å) radiation for the same single crystal of **1** at 150, 229 and 300 K. Data processing was performed by using the SAINT processing program. The crystal structure was solved through direct methods, and all the non-hydrogen atoms were refined with anisotropic thermal parameters on *F*^2^ by full-matrix least-squares, using the SHELXTL 2014 and Olex 2 program. The detailed crystallographic data and structure refinement parameters were summarized for **1** (CCDC No. 2,131,162 for 150 K, 2,131,163 for 229 K and 2,131,164 for 300 K) in Supplementary Materials Tables S1 and S2. The crystallographic data can be obtained free of charge from www.ccdc.cam.ac.uk/data_request/cif (accessed on 27 December 2021).

### 3.3. Synthesis of Abtz

A total of 30 mL anhydrous EtOH of thiazole-4-carbaldehyde (11.0 mmol, 1.24 g) was added dropwise into a 50 mL anhydrous EtOH of 4-aminoazobenzene (10.0 mmol, 1.97 g). Subsequently, the mixture was stirred and heated at 80 °C under refluxing for 8 h. After removing the solvent, the dark yellow powder of Abtz (2.74 g, yield: 93.7%) was obtained. ^1^H NMR (400 MHz, DMSO): 9.28(s, 1H), 8.80(s, 1H), 8.51(s, 1H), 7.98 (d, 2H), 7.92(d, 2H), 7.60(m, 3H), 7.50(d, 2H).

### 3.4. Synthesis of Fe(Abtz)_3_

A 3 mL ethyl acetate solution of Fe(BF_4_)_2_·6H_2_O (0.4 mmol, 13.5 mg) was added in a 5 mL vial, and a dissolved Abtz solution (0.1 mmol, 29.2 mg) with ethyl acetate (5 mL) was added in 40 mL glass bottle. The vial was put into the glass bottle and then slowly added dropwise ethyl acetate until the liquid level was higher about 3 cm than the inner vial. After sealing in static conditions for one week, the red block crystals for compound **1** with the composition C_56_H_52_B_2_F_8_FeN_12_O_4_S_3_ were obtained. Anal. calcd. for C_56_H_52_B_2_F_8_FeN_12_O_4_S_3_ (%): C, 52.43; H, 4.09; N, 13.10. Found: C, 52.40; H, 4.14; N, 13.00.

## 4. Conclusions

In summary, inspired by the strategy of utilizing photochromic ligand to construct photoresponsive SCO materials, we synthesized a new chelating ligand, Abtz, which is decorated by azobenzene group. The potential photoisomerization behaviors for ligand Abtz and the corresponding SCO molecule in both solid state and liquid state were completely studied. Under continuous UV and blue-light irradiation, the azobenzene group of Abtz in liquid undergoes successful reversible *cis–trans* photoisomerization behavior, while the solid-state photoisomerization behavior was not observed. Moreover, the mononuclear Fe(II) molecule **1** based on this photochromic ligand shows a one-step SCO behavior in the temperature range of 150–300 K. However, due to the possible decomposition in liquid state and steric hindrance in the solid state, the potential photoisomerization of crystalline samples for **1** is unsuccessful. To obtain the solid-state LD-LISC behavior, a dispersion of molecules may be the key to improving the photoisomerization process. Therefore, loading of LD-LISC molecules in molecular sieves, which show the adjustable pore size, may be a desirable strategy to realize improved solid-state LD-LISC behavior. In fact, some excellent works have demonstrated that the loading of azobenzene units or azobenzene-decorated molecules into molecular sieves could obviously improve the reversible *cis–trans* photoisomerization behavior, thus giving the reversible controlling of functional properties, such as gas absorption and birefringence [32,33]. However, the decoration of LD-LISC molecules in molecular sieves was not reported, as far as we know. Further attempts on the dispersion of **1** in the molecular sieve would be performed to increase the free space between molecules to realize highly effective photoisomerization in the future.

## Figures and Tables

**Table 1:** Bond lengths of Fe-N for [Fe(Abtz)_3_]^2+^ at 150, 229 and 300 K.

Bond Lengths/Å	150 K	229 K	300 K
Fe-N1	1.977(2)	2.067(3)	2.178(4)
Fe-N2	2.004(2)	2.096(3)	2.199(4)
Fe-N3	1.982(2)	2.067(3)	2.160(4)
Fe-N4	2.016(2)	2.130(3)	2.252(4)
Fe-N5	1.960(2)	2.035(3)	2.116(4)
Fe-N6	2.040(2)	2.134(3)	2.242(4)
<Fe–N> ^a^	1.997	2.091	2.191

^a^ Average Fe–N bond lengths.

## Data Availability

All the relevant data have been presented in this published article.

## Supplementary Materials

The following Supporting Materials are available online. Table S1: Crystal data and structure refinement for **1** at 150, 229 and 300 K. Table S2: Bond angles of N-Fe-N for **1** at 150, 229 and 300 K. Figure S1: A view showing the unit cell of **1** along the axis. Figure S2: A view showing the supramolecular interaction between [Fe(Abtz)_3_]^2+^ molecules. Figure S3: A comparison of the molecule structures of **1** at 150 and 300 K. Figure S4: A comparison of fingerprint plots for **1** at 150 and 300 K.

## Sample Availability

Samples of the compounds are available from the authors.
