# Supplementary material for: A Mononuclear Iron(II) Spin-Crossover Molecule Decorated by Photochromic Azobenzene Group
Source: Molecules. 2022 Feb 27;27(5):1571. doi: 10.3390/molecules27051571 (PMC8912052; doi:10.3390/molecules27051571)
Supplement: Supplementary file 1 [file molecules-27-01571-s001.zip › molecules-1554622-supplementary.pdf]

Supporting Information

A Mononuclear Iron(II) Spin-Crossover Molecule Decorated by Photochromic Azobenzene Group

Jiang-Zhen Qiu [1,*], Yong You [1], Ye Yu [1], Zhuo-Fan Chen [1], Cheng-Jie Guo [1], Yi-Ling Zhong [2], Wei-Quan Lin [2,*], and Xu-Gang Shu [1,*]

1 College of Chemistry and Chemical Engineering, Zhongkai University of Agriculture and Engineering, Guangzhou 510225, P. R. China.

2 School of Chemistry and Chemical Engineering/Guangzhou Key Laboratory for Clean Energy and Materials, Guangzhou University, Guangzhou 510006, P. R. China.

* Correspondence: qjz_qiu@126.com (J.-Z. Q.); xgshu@21cn.com (X.-G. S.); linwquan@gzhu.edu.cn (W.-Q. L.)

Table S1. Crystal data and structure refinement for **1** at 150 K, 229 K, and 300 K.

**Compound**		**1**	
***T* [K]**	150	229	300
**Formula**		$C_{56}H_{52}B_2F_8FeN_{12}O_4S_3$	
**Formula weight**		1282.74	
**Crystal system**		triclinic	
**Space group**		*P-1*	
***a* / Å**	12.4246(6)	12.6311(9)	12.8311(10)
***b* / Å**	13.9971(6)	14.0287(8)	14.0706(10)
***c* / Å**	18.4217(9)	18.5265(12)	18.6133(14)
***α* / °**	104.6960(10)	105.475(2)	105.731(2)
***β* / °**	102.711(2)	101.820(2)	100.936(2)
***γ* / °**	96.930(2)	97.504(2)	98.172(2)
**Volume / Å^3**	2969.6(2)	3036.5(3)	3108.5(4)
***Z*, ρ_{calcg} / cm^3**	2, 1.435	2, 1.403	2, 1.370
**μ / mm^{-1}, F(000)**	0.441, 1320.0	0.432, 1320.0	0.422, 1320.0
**Wavelength / Å**	0.71073	0.71073	0.71073
**Refl. Coll.**	62150	63359	58210
**Goodness of Fit on F^2**	1.032	1.02	1.057
**R_1 [I > 2σ (*I*)]**	R_1 = 0.0532	R_1 = 0.0797	R_1 = 0.0919
**R_2 [all data]**	wR_2 = 0.1400	wR_2 = 0.2339	wR_2 = 0.2890

$R_1=\sum||F_o|-|F_c||/\sum|F_o|$. $wR_2 = [\sum[w (F_o^2-F_c^2)^2] / \sum[w (F_o^2)^2]]^{1/2}$.

Table S2. The bond angles of N-Fe-N for **1** at 150 K, 229 K, and 300 K.

Bond Angles / °	150 K	229 K	300 K
N1-Fe1-N2	81.12(8)	79.00(12)	76.78(14)
N1-Fe1-N3	88.58(8)	90.25(13)	92.01(16)
N1-Fe1-N4	93.98(8)	95.57(12)	97.20(14)
N1-Fe1-N5	93.28(9)	93.35(13)	93.85(16)
N2-Fe1-N3	93.15(8)	92.36(13)	91.92(15)
N3-Fe1-N4	80.81(8)	78.65(12)	76.00(15)
N4-Fe1-N5	92.20(8)	91.33(12)	90.40(15)
N5-Fe1-N2	93.93(8)	97.86(13)	102.03(15)
N6-Fe1-N2	99.52(8)	101.37(12)	103.58(13)
N6-Fe1-N3	96.90(8)	97.19(12)	97.34(15)
N6-Fe1-N4	85.92(8)	85.19(11)	84.36(14)
N6-Fe1-N5	81.16(8)	79.19(12)	76.86(15)
ΣFe[a]	65.37	77.25	80.75

[a]Octahedral distortion parameters (°)

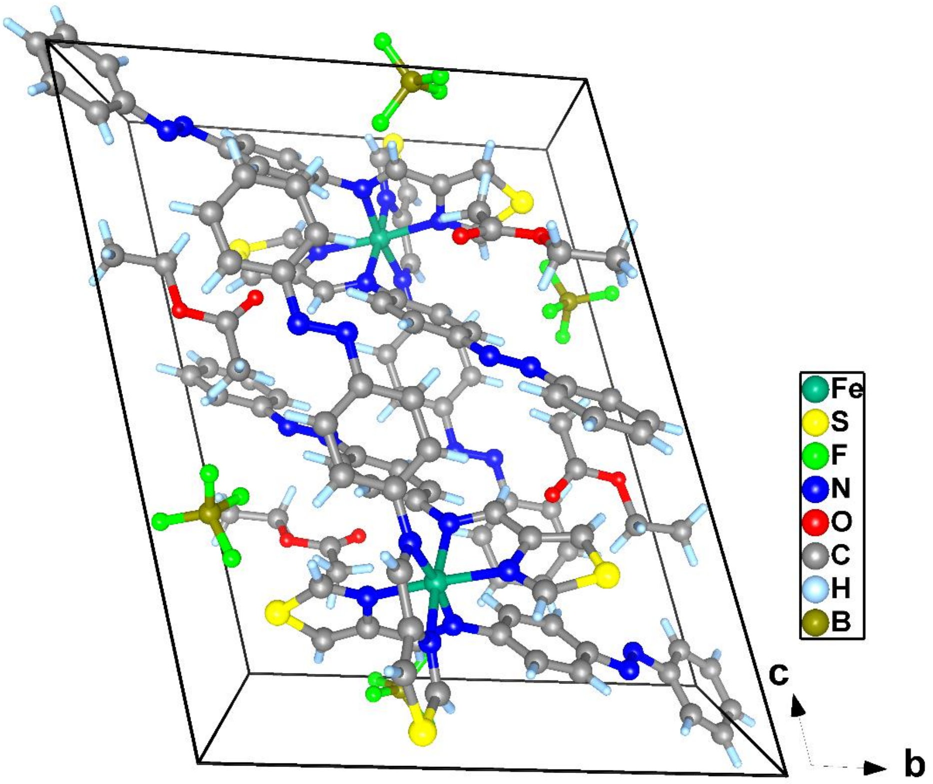

Figure S1. A view showing the unit cell of **1** along the *a* axis.

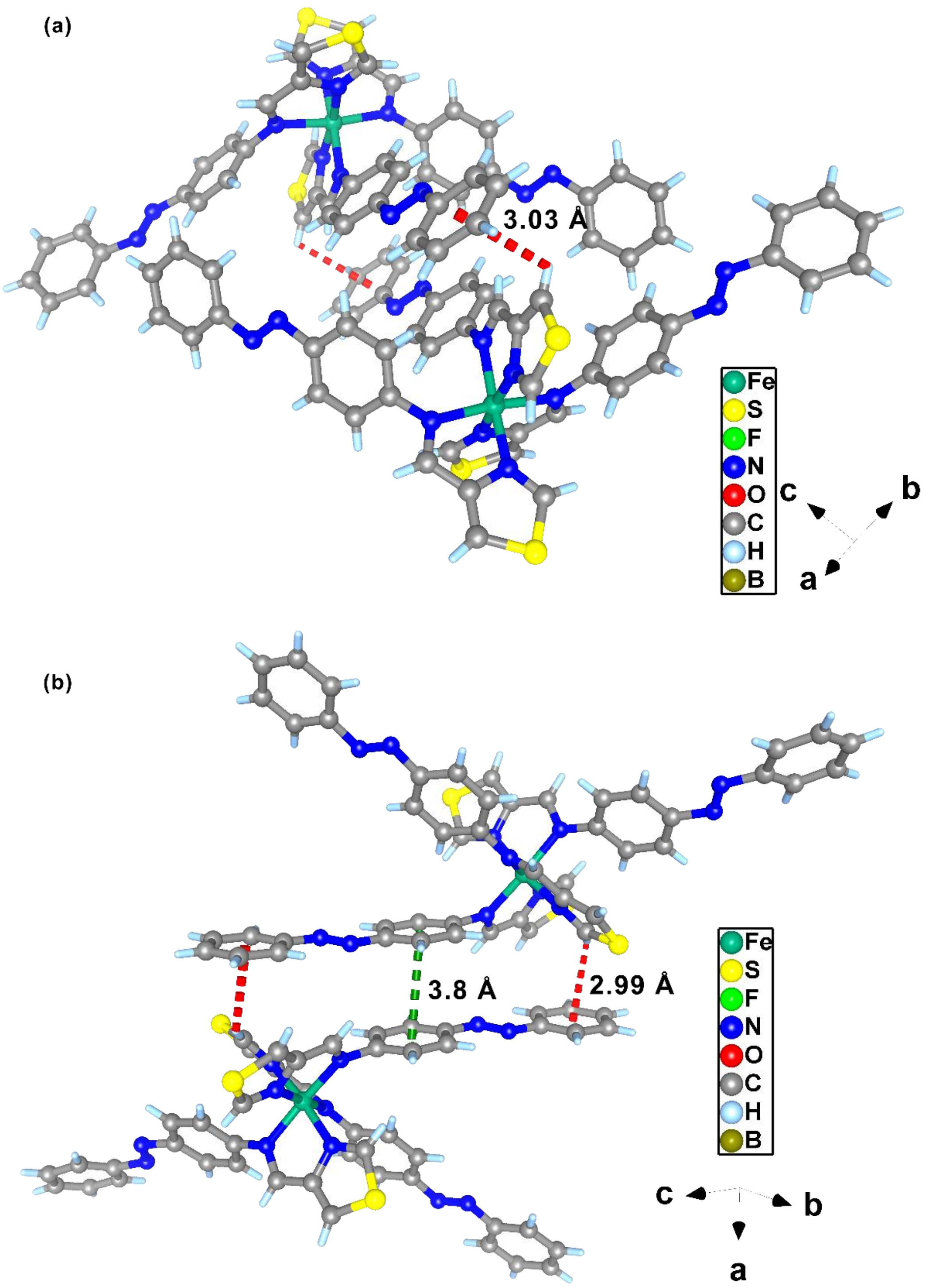

Figure S2. A view showing the supramolecular interaction between $[Fe(Abtz)_3]^{2+}$ molecules, the offset face-to-face $\pi...\pi$ interactions and edge-to-face $C\text{-}H...\pi$ interactions are displayed as red and green dashed lines, respectively.

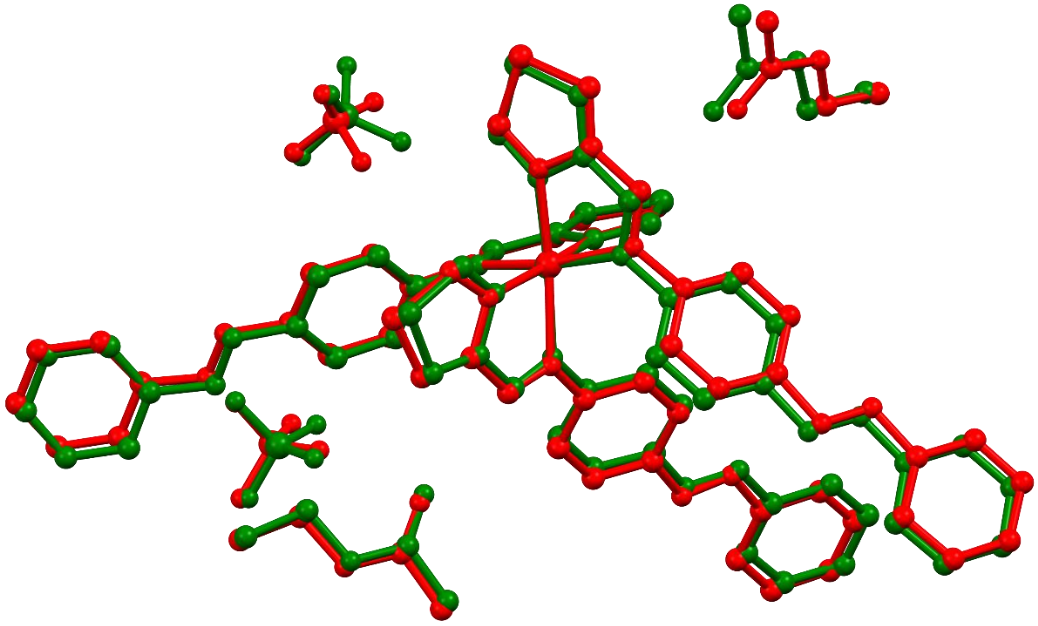

Figure S3. A comparison of the molecule structures of **1** at 150 K (green) and 300 K (red), showing the rotation of BF_4^- anions during the SCO process.

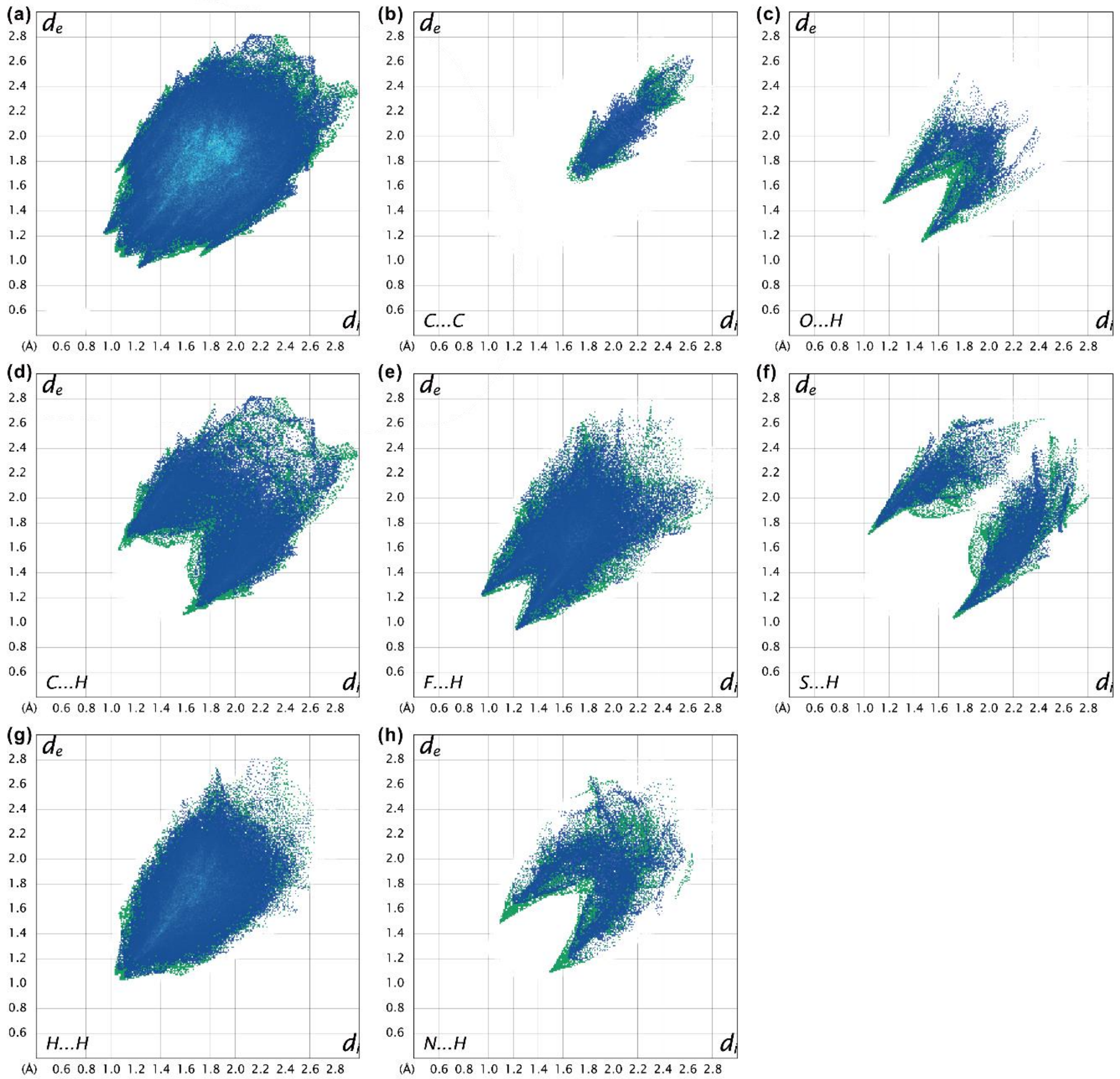

Figure S4. A comparison of fingerprint plots with all intermolecular interactions (a) resolved into the contribution of C···C (b), O···H (c), C···H (d), F···H (e), S···H (f), H···H (g), and N···H (h) contacts of complex **1** at 150 K (green) and 300 K (blue).
